# Supplementary material for: Multi-trajectory analysis of changes in physical activity and body mass index in relation to retirement: Finnish Retirement and Aging study
Source: PLoS One. 2022 Dec 1;17(12):e0278405. doi: 10.1371/journal.pone.0278405 (PMC9714751; doi:10.1371/journal.pone.0278405)
Supplement: S1 Table — (PDF) [file pone.0278405.s001.pdf]

Supplement table S1. Physical activity and body mass index at different waves by trajectory groups

Study waves	Group 1. Stable normal weight and high physical activity (n=1,068)			Group 2. Stable overweight and moderate physical activity (n=1,307)			Group 3. Stable class I obesity and moderate physical activity (n=769)			Group 4. Stable class II obesity and low physical activity (n=207)		
	Mean	95% CI		Mean	95% CI		Mean	95% CI		Mean	95% CI	
Physical activity, MET/week												
-2	31.4	29.6	33.7	24.9	23.7	27.6	19.6	18.5	23.3	10.5	9.9	17.4
-1	30.0	29.6	32.6	23.3	23.2	26.0	18.6	19.0	22.4	11.1	11.8	17.2
1	32.7	30.9	33.4	25.3	24.4	26.9	21.0	20.3	23.2	13.4	13.6	18.4
2	32.1	31.7	34.9	25.4	25.1	28.2	20.3	20.5	24.3	13.7	13.2	19.8
3	33.2	30.5	35.7	24.7	22.9	27.9	19.9	17.9	24.3	11.2	9.4	18.8
Body mass index, kg/m2												
-2	22.3	22.2	22.5	26.2	26.0	26.3	30.7	30.5	30.9	37.1	36.7	37.4
-1	22.4	22.3	22.5	26.3	26.2	26.5	30.9	30.8	31.1	37.4	37.2	37.7
1	22.4	22.3	22.5	26.4	26.2	26.5	30.9	30.7	31.0	37.7	37.4	37.8
2	22.3	22.2	22.5	26.4	26.2	26.5	30.8	30.6	31.0	37.4	37.2	37.8
3	22.5	22.3	22.6	26.5	26.3	26.7	30.9	30.6	31.2	37.2	36.7	37.6
